# S100B as an antagonist to block the interaction between S100A1 and the RAGE V domain

**DOI:** 10.1371/journal.pone.0190545

**Published:** 2018-02-14

**Authors:** Md. Imran Khan, Yu-Kai Su, Jinhao Zou, Lee-Wei Yang, Ruey-Hwang Chou, Chin Yu

**Affiliations:** 1 National Tsing Hua University, Chemistry Department, Hsinchu, Taiwan; 2 Institute of Bioinformatics and Structural Biology, National Tsing Hua University, Hsinchu, Taiwan; 3 Physics Division, National Center for Theoretical Sciences, National Tsing Hua University, Hsinchu, Taiwan; 4 Graduate Institute of Biomedical Sciences, China Medical University, Taichung, Taiwan; 5 Center for Molecular Medicine, China Medical University Hospital, Taichung, Taiwan; 6 Department of Biotechnology, Asia University, Taichung, Taiwan; Russian Academy of Medical Sciences, RUSSIAN FEDERATION

## Abstract

Ca2^+^-binding human S100A1 protein is a type of S100 protein. S100A1 is a significant mediator during inflammation when Ca^2+^ binds to its EF-hand motifs. Receptors for advanced glycation end products (RAGE) correspond to 5 domains: the cytoplasmic, transmembrane, C2, C1, and V domains. The V domain of RAGE is one of the most important target proteins for S100A1. It binds to the hydrophobic surface and triggers signaling transduction cascades that induce cell growth, cell proliferation, and tumorigenesis. We used nuclear magnetic resonance (NMR) spectroscopy to characterize the interaction between S100A1 and the RAGE V domain. We found that S100B could interact with S100A1 via NMR ^1^H-^15^N HSQC titrations. We used the HADDOCK program to generate the following two binary complexes based on the NMR titration results: S100A1-RAGE V domain and S100A1-S100B. After overlapping these two complex structures, we found that S100B plays a crucial role in blocking the interaction site between RAGE V domain and S100A1. A cell proliferation assay WST-1 also supported our results. This report could potentially be useful for new protein development for cancer treatment.

## 1. Introduction

Human S100 proteins are Ca^2+^-binding low-acidic proteins (10–13 kDa) with more than 20 family members in vertebrates [1]. These proteins are deposited as a cluster on human chromosome 1q21 [2]. They constitute the largest subfamily of EF-hand (helix-loop-helix structure) Ca^2+^-binding proteins. They are well known to mediate intracellular Ca^2+^ signals by an unknown pathway [3,4]. The S100 protein family has an unprotected hydrophobic binding pocket that mediates protein-protein interactions [5–7]. Most S100 proteins have the ability to form homodimers and some heterodimers [8], and they appear to form advanced types of homo- and heterooligomers [9,10]. Oligomerization is a common mechanism after the activation of cell surface receptors, and many proteins are known to oligomerize, such as Ig- [11].

S100A1 protein was first detected in the brain and is expressed in the heart, thyroid gland, skin, skeletal muscles, salivary glands, kidneys, ovaries, and breasts. It causes neurodegenerative disorders, various cancers (thyroid, breast renal, melanoma, endometrial, and endometrioid cancers), and other diseases [12–26]. Cardiomyopathy altered the expression of S100A1 [21]. Upon calcium binding, the conformation of helix 3 and helix 4 on S100A1 results in reorientation that exposes a large hydrophobic pocket between these helices, and most calcium-dependent target proteins interact with this region [27]. Previous studies show that S100A1 could interact with other proteins like RAGE, RyR2, TRPM3, ATP2A2, and RyR1 [28–33]. It also induces the activities or conformational changes of S100A1, which promote specific physiological functions. S100A1 plays an important role in heart failure, and S100A1 gene therapies were recently implemented in the human clinical trials [34].

RAGE (MW 35 kDa) is a part of the immunoglobulin (Ig) family [35,36] and consists of five domains: one cytoplasmic domain for signal transduction at the C-terminal, one transmembrane domain that anchors the receptor to the membrane, two distinct constant domains (C1, C2), and one variable domain (V) at the N-terminal [37,38]. The V domain of RAGE is the primary receptor involved in binding ligands, such as proteins of the S100 family and advanced glycation end products (AGEs). The mechanisms by which some S100 family proteins bind to RAGE are still unclear. Once ligands bind to RA GE, tail-like cytoplasmic domains become homo-dimerized with various charged residues. There are correlated with autophosphorylation and promote intracellular downstream signal transduction, which triggers various diseases, such as diabetes [39] neurodegeneration [40], chronic vascular inflammation, and cancers [38]. These signal transductions induce inflammation, cell proliferation, migration, and even tumor growth [41,42]. However, the pathway that will be activated depends on the type and concentration of binding ligands in different cells. Therefore, insight into the interaction of RAGE with its targets and signal pathways are important for improving or developing treatments for RAGE-dependent diseases [43].

The V domain is the primary receptor for S100 protein binding [33]. Some S100 proteins, such as S100A6 [44] and S100A12 [45], can interact with variable types of the V domain simultaneously through their constant domains (C1 or C2). To date, it is still unknown how the S100 protein family binds to RAGE [33]. S100A1 was reported to bind to the V domain of RAGE in an SPR experiment [33] and induce RAGE autophosphorylation, signal transduction cascade, and cell proliferation [46]. The interaction between S100A1 and RAGE V domain was reported several years ago, but not at the molecular level. In this study, we examined the interface among S100A1 and the V domain and constructed a S100A1-V domain heterodimeric complex to demonstrate the interface at the molecular level. The S100A1 molecule contains a free cysteine at residue 85. The cysteine leads to the formation of intermolecular disulfide bonds, which affect the accuracy of NMR spectra [47]. Thus, 5mM of dithiothreitol (DTT) was needed as a reducing agent in the NMR ^1^H-^15^N HSQC titration experiments to prevent the oxidation of cysteine residues to form disulfides. However, DTT can break disulfide bonds in the V domain. This problem was solved by mutating cysteine to serine (C85S) on S100A1 [48]. This method can be used to replace S100A1 with mutant-S100A1 (C85S) for NMR experiments without using DTT. We generated the mS100A1-RAGE V domain and S100A1-S100B heterodimeric complex structure using the HADDOCK program [49,50]. Residues where chosen on each protein interface based on the chemical shift perturbation from the NMR ^1^H-^15^N HSQC titration results. All model structures were illustrated using PyMOL [51].

S100B is the first described member of the Ca^2+^-binding S100 protein family and the most widely examined. It has been reported that S100B plays an extracellular role in types of many cells, such as arterial smooth muscle cells, adipocytes, melanocytes, Schwann cells, chondrocytes, microglia, and particularly astrocytes [52]. Adjacent to helix 3 of S100B, Ca^2+^-binding exposes a huge hydrophobic groove that can hold interacting proteins and peptides. The result can be confusion, memory loss, and brain disorder through Alzheimer’s disease (AD) [53–59]. S100B is released by neuroglial cells in AD patients, and higher concentrations have been found in the cerebrospinal fluid and fluent serum [60,61]. The action of S100B is stronger in the pathogenesis of neurodegenerative progressions [59,62]. Depending on the concentration, S100B can potentially have both neurotoxic and neurotrophic outcomes [63]. The overexpression of S100B implies effects on the movement of distributed nonfibrillar amyloid deposits to neurotic forms and accordingly on the development of the disease [59]. Additionally, the serum levels of S100B can be useful for assessing the harshness of AD or to detecting the growth of dementia [55]. Hence, S100B can be used as a possible biochemical marker of AD development [55,59,62,64–66].

S100A1 and S100B are homogeneously expressed in all zones of normal articular cartilage [67] but are lost in osteoarthritic (OA) cartilage, together with proteoglycans and type II collagen [68]. The expression of S100A1 and S100B is high in human articular chondrocytes compared to osteophytic chondrocytes [69]. Few studies address the intracellular roles of S100 proteins in human articular chondrocytes (HAC), but they strongly suggest an important role of S100A1 and S100B in chondrocyte biology. S100A1 and S100B are the first members of the S100 protein family to be discovered and are the best-characterized. They also have a high degree of sequence and structural similarity. S100A1 and S100B are highly expressed and studied in the heart and brain, respectively [25]. We used S100B as an antagonist to block the interaction between S100A1 and the V domain, thus inhibiting signal transduction. We also demonstrate that the inhibition properties of S100B by WST-1 cell proliferation assays [70]. Finally, we provide putative models of S100A1 in complex with a S100B molecule and the V domain, which may provide significant insights for future disease treatments.

## 2. Materials and methods

### 2.1. Materials

Isotopic-labeled deuterium oxide (D_2_O, 99%) and ^15^N-ammonium chloride (^15^NH_4_Cl, 99%) were obtained from Cambridge Isotope Laboratories. Milli-Q water was used for preparation of all solutions. All the sample buffers for NMR spectroscopy were cleaned using a 0.22-μm sterile filter. SW-480 cells were obtained from the American Type Culture Collection (CCL-288).

### 2.2. Preparation of S100A1

The recombinant cDNA of protein sequence (1–93) of S100A1 was inserted in NdeI or XhoI restriction sites. A pET-20b bacterial expression vector was then used for cloning, which was transferred and expressed in Novagen Rosetta^™^ (DE3), a BL21 derivative designed to enhance the expression [71,72]. ^15^N-labeled S100A1 protein was prepared by culturing *E*. *coli* containing the S100A1 gene in M9 medium [73]. When the optical density (O.D.) reached 0.8–1.0 at 600 nm, 1 mM isopropyl β-D-1-thio-galacto-pyranoside (IPTG) was added to cultures induced at 200 rpm in incubators at 37°C for 10 hr.

The cells were harvested by centrifugation of 30 min at 6000 rpm. The cells were lysed for 1 h using a sonicator in a resuspension buffer consisting of 20 mM Tris-HCl and 1 mM ethylene diamine tetra AcOH (EDTA) at pH 7.5. Most of the proteins were in the supernatant fraction after centrifuging at 12000 rpm for 40 min at 4°C. The supernatant containing S100A1 was filtered and purified using a Hi-Prep Phenyl FF 16/10 Q-Sepharose column on a GE Healthcare FPLC system. The fraction containing S100A1 was eluted with a gradient 0–1.0 M of sodium chloride (NaCl). Then, the S100A1 fraction was transferred to a buffer containing 100 mM NaCl, 10 mM calcium chloride (CaCl_2_), and 20 mM Tris-HCl at pH 7.5, which was purified by a Hi-Prep Phenyl FF 16/10 hydrophobic interaction chromatography (HIC) column on the AKTA FPLC system.

The pure S100A1 protein was collected by gradient elution with buffer including 20 mM EDTA and 20 mM Tris-HCl at pH 7.5. As a final purification step, the protein was placed in NMR buffer containing 20 mM CaCl_2,_ 10% D_2_O, 15 mM NaCl, and 20 mM Tris-HCl at pH 7.5 in a millipore centrifuge tube. The samples were pure (S1 Fig).

### 2.3. Expression and purification of RAGE V domain

The pET-32b (+) bacterial expression vector was used to clone the recombinant RAGE V domain and transferred into Rosetta^™^ (DE3). The expression of ^15^N-labeled RAGE V-domain protein was similar to that of S100A1 the when O.D. of the culture reached 0.6–0.7 at 600 nm than induced with IPTG at 200 rpm and 25°C for three hours. The cells were harvested by centrifugation at 6000 rpm for 30 min. They were then lysed using a sonicator for 1 h in resuspension buffer consisting of 300 mM NaCl and 20 mM Tris-HCl at pH 8.0.

Most of the proteins were in the mother liquid fraction after centrifuging at 12000 rpm for 40 min 4°C. The supernatant fraction was filtered and loaded into a Nickel Sepharose 6 Fast Flow (GE Healthcare) Immobilized Metal Affinity Chromatography (IMAC) column. The RAGE V domain binds to Ni^2+^ with its six adjacent histidines on a His tag at the N-terminus. The V domain was eluted with 500 mM imidazole in resuspension buffer. The RAGE V domain was cleaved from the His tag with thrombin digestion for 3 h at 25°C. It was then purified further by HPLC (HITACHI L-7000) with an Atlantis dc18 Prep Column (Waters) using a gradient of 45% acetonitrile and 0.1% trifluoro acetic acid over 42 min. We used SDS-PAGE to confirm that the samples were pure (S2 Fig).

### 2.4. Preparation of S100B

For the protein expression, human S100B cDNA of 1–91 amino acids was cloned in vector pET-20b (+) with BL21 host cells (DE3). Cultures were allowed to grow at 37°C to an OD of 0.8–1.0. Then, 1.0 mM IPTG was used for induction, and the culture was allowed to grow at 37°C for 6–8 h. The buffer used for resuspension contained 2 mM DTT, 1 mM EDTA, and 20 mM Tris/HCl (pH 7.5), which was used to break the cells with a sonicator.

After centrifugation at 12000 rpm at 4°C for 40 min, the supernatant fraction contained most of the S100B protein. The supernatant was purified by a GE Healthcare Q-sepharose column, and the S100B protein was eluted with a buffer gradient containing 0–1.0 M NaCl. The concentrated elute of the sQ-column fraction was purified by FPLC Pharmacia Superdex 75 size exclusion chromatography column using 5 mM CaCl_2_, 50 mM (NH_4_)_2_SO_4,_ 5 mM DTT, and 20 mM Tris/HCl (pH 7.2). The purified S100B was confirmed by SDS-PAGE (S3 Fig).

### 2.5. NMR ^1^H-^15^N HSQC titration experiments

NMR HSQC titrations were carried out at 25°C using a cryogenic probe within a Varian 700 MHz spectrometer. The same buffer was used for all samples and contained 20 mM Tris buffer (in 10% D_2_O: 90% H_2_O), 5 mM CaCl_2_, 5 mM DTT, and 50 mM (NH_4_)_2_SO_4_ at pH 7.2. Next, ^1^H-^15^N HSQC experiments titrations were performed by adding unlabeled RAGE V domain to samples of ^15^N**-**labeled S100A1 at molar ratios of 1:0 and 1:1.

We also performed a different titration using the ^15^N**-**labeled RAGE V domain by adding unlabeled S100A1 at molar ratios of 1:0 and 1:1. The resulting HSQC spectra were superimposed to determine whether the cross-peaks were shifted or whether the intensities were severely decreased. For the S100B–S100A1 titration, ^15^N**-**labeled S100B protein was titrated using molar ratios of 1:0 and 1:1 with unlabeled S100A1 protein, and vice versa. The spectra were examined using Sparky [74].

### 2.6. Biomolecular docking (HADDOCK)

We used HADDOCK (version 2.2) to construct models of the S100A1-V domain complex and S100A1-S100B complex. NMR solution structures of the Ca^2+^-bound S100A1 homodimer were obtained from the Protein Data Bank (PDB ID: 2LP3). The V domain and S100B [75] structures were also obtained from the PDB (ID: 2E5E and 1UWO, respectively). The docking and subsequent refinement were done in the presence of a number of ambiguous interaction restraints (AIRs), applied at the residues that correspond to HSQC-identified cross-peaks with significant chemical shifts or decreased intensities. Among these residues, we considered the ones with a higher solvent accessibility to serve as the main interface residues. Therefore, those have a relative solvent accessibility (RSA) higher than 30% are defined as the "active residues" while those lower than 30% are the "passive residues" for the parameterization of HADDOCK. Here, RSA was calculated by the software NACCESS [76]. According to HADDOCK, active residues should be part of the interface while passive residues can also be at the interface, but will not be energetically penalized if they are not.

The central idea of the HADDOCK is to use experimentally determined contact information (including but not limited to those from NMR, mutagenesis data, chemical crosslinking etc) to guide the docking and molecular simulation-based (MD-based) refinement [49,50]. Each resulting S100A1-S100B complex, or, "docking pose" is associated with a weighted sum of energy comprising terms of van der Waals, electrostatic, desolvation, restraint violation (the energetic penalty for a complex’s violation of the experimental data) and buried surface area (with a negative weight) based on OPLS forcefield [77] such that

$${\text{HADDOCK}}_{\text{SCORE}}=1.0^{*}{\text{E}}_{\text{vdw}}+0.2^{*}{\text{E}}_{\text{elec}}+1.0^{*}{\text{E}}_{\text{desol}}+0.1^{*}{\text{E}}_{\text{air}}$$

where E_vdw_ is the intermolecular van der Waals energy, E_elec_ is the intermolecular electrostatic energy, E_desol_ represents an empirical desolvation energy term adapted from Fernandez[78] and E_air_ is the energy imposed by ambiguous interaction restraint (AIR).

Initially, about 5000 complex structures that resulted from rigid-body (not flexible) docking were collected. The interface residues (containing heavy atoms within 5 Angstrom away from the other protein) of the "best" 200 complexes, having the lowest HADDOCK score (or energy) among the 5000, were subject to semi-flexible simulated annealing in the torsion angle space, followed by short MD simulations in explicit solvent (TIP3P water model was used in an 8-Angstrom hydration shell). The final 200 refined structures were further grouped into 10 clusters based on the interface similarity between the constituent docking poses. The clusters were then rank-ordered per their average energies of the 4 members that have the lowest energies in each cluster, according to the default setting of HADDOCK. The reported complexes in this study were taken from the first cluster, displayed and illustrated using PyMOL [53].

### 2.7. WST-1 assay analysis

The physiological effect of S100 proteins in cells was determined by WST-1, 2-(4-nitrophenyl) 4-[3-(4-iodophenyl)-2H-5-tetrazolio]-1,3-benzene disulfonate), cell proliferation assay [79]. WST-1 is cleaved to a soluble formazan by mitochondrial dehydrogenases in the living cells. The amount of formed formazan directly correlates to enzymatic activity of dehydrogenases, which is positively associated with the number of metabolically active living cells in the culture. SW480 cells (ATCC: CCL-228) were placed in a 96-well plate at a density of 5 × 10^3^ cells/well one day before the experiments. The cells were then incubated for a day without serum in a medium containing 0.1% BSA. The serum-starved cells were treated with 10, 50, or 100 nM S100A1 protein with or without 100 nM S100B protein for another 48 h to observe the gradient change of the relative cell number. Before harvesting, 10 μL of WST-1 reagent was added into individual well containing 100 μL cell culture medium, and cells were incubated for another 4 h at 37°C. The cell culture plate was shaken for 10 min with mild agitation on a shaker. The absorbance was determined at an optical wavelength of 450 nm using a Synergy 2 microplate reader (BioTek Instruments, VT, USA). The relative cell numbers were determined by comparing the relative absorbance to that of the control treatment.

## 3. Results and discussion

### 3.1. The-binding interface of S100A1 and V domain

**Fig 1:**
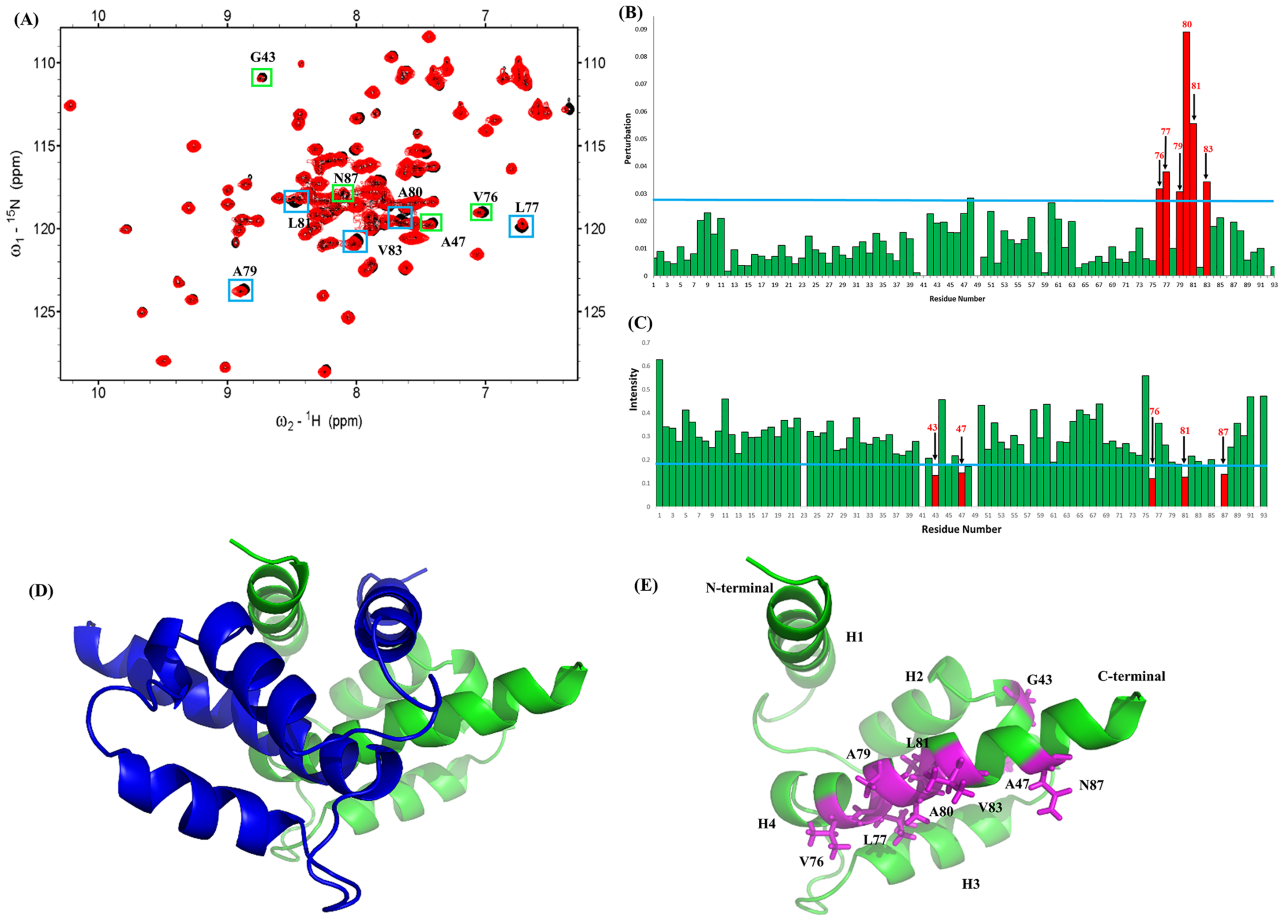
Analysis of the ^1^H–^15^N HSQC spectra of S100A1 in complex with the unlabeled RAGE V domain. (A) Superimposed ^1^H-^15^N HSQC spectra of ^15^N S100A1 (in black) and ^15^N S100A1 in complex with unlabeled RAGE V domain (red). Cross-peaks display significantly decreased intensity, and those with perturbed chemical shifts are boxed in green and blue, respectively. (B) Bar plot showing the chemical shift variations in cross-peaks for S100A1 and the S100A1-V domain complex. The threshold (blue line) of the chosen residues (red) displays a notable perturbation. (C) Bar graph analysis representing the change in intensities (I/I_0_) of cross-peaks of free ^15^N S100A1 to ^15^N S100A1 in complex with V domain against residue numbers of S100A1 (1–93). I_0_ denotes the initial intensity of free ^15^N S100A1, and I denotes the intensity of ^15^N S100A1 in complex with V domain. The blue line represents the criterion of selected residues that exhibit a significantly decreasing cross-peak signal, and the chosen residues are displayed in red bars. (D) Ribbon representing the structure of homo-dimer. The monomers of S100A1 homodimer are colored in green and blue. (E) A monomer of S100A1 and residues that exhibit significantly decreasing cross-peak signals are mapped on the structure in magenta.

NMR ^1^H-^15^N HSQC experiments are broadly used to identify binding interfaces between proteins or between proteins and ligands. The residues of S100A1 and RAGE V domain at their interfaces can be identified by observing the resonances on the NMR HSQC spectra of S100A1 and comparing them with those of the S100A1 in complex with the V domain. Fig 1A shows the superimposed HSQC spectra of free ^15^N-labeled S100A1 and ^15^N-labeled S100A1 in complex with unlabeled V domain. Parts of the NMR signals were decreased when adding free RAGE V domain to ^15^N-labeled S100A1. The NMR signals of the S100A1 complex with V domain constraints at the interface were obviously significantly lower than that obtained with free S100A1. This phenomenon was due to the affected nuclei at the-binding sites of the proteins between S100A1 and V domain.

The surrounding of nuclei at the interface were changed by the other protein being closed and led to lower cross-peaks on the NMR spectrum. The residues of S100 proteins were affected by complex formation with S100 target proteins. Previous NMR studies pointed out changes in NMR resonance between protein-protein complex interfaces. Therefore, the H^1^-N^15^ HSQC spectra of free S100A1 and S100A1 in complex with the V domain were overlapped to identify the perturbation of S100A1 residues (V76, L77, A79, A80, L81 and V83) at the interface.

A bar graph is shown in Fig 1B for comparison of the residues from both spectra. The bar graph was used to determine the residues of S100A1 that interact with the V domain by comparing the HSQC cross-peaks that shifted between free S100A1 (I_0_) and its complex with the V domain (I). Variations in the cross-peak chemical shifts were used to compare free S100A1 (N_0_, H_0_) to the complex with unlabeled S100B (N, H). The formula for the chemical shift variation is:

$$\Delta \delta =\sqrt{{\left(\delta^{1}\text{HN}\right)}^{2}+0.1{\left(\delta^{15}\text{N}\right)}^{2}}$$ (1)

This equation for the perturbation calculation was derived by an NMR spectroscopy research group of Utrecht University [80].

Some residues of the HSQC cross-peaks showed significantly lower intensity in the bar diagram (Fig 1C). These S100A1 residues were G43, A47, V76, L81, and N87. The residues were chosen for mapping on the 3D structure of the S100A1 domain (Fig 1D).

### 3.2. Binding interface of the V domain and S100A1

**Fig 2:**
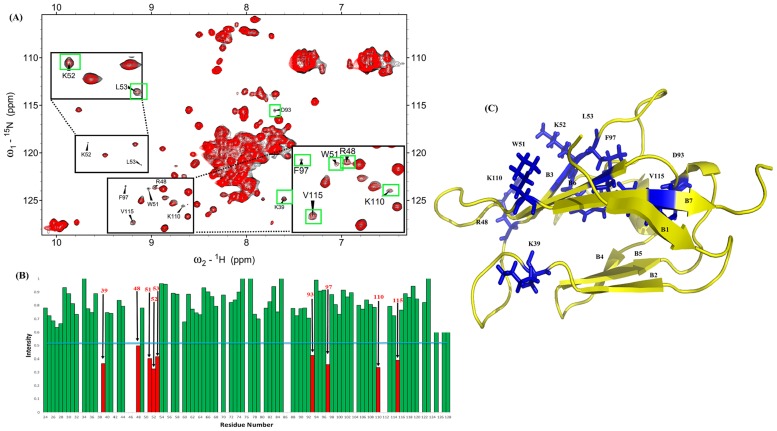
Analysis of the ^1^ H–^15^N HSQC spectrum of the RAGE V domain with unlabeled S100A1. (A) The superimposed ^1^H-^15^N HSQC spectra of ^15^N V domain in black and ^15^N V domain complex with S100A1 in red are in a molar ratio of 1:1. Cross-peaks displaying significantly decreased intensity are shown in green boxes. (B) Bar graph analysis representing the intensity change (I/I_0_) of cross-peaks of ^15^N V domain to ^15^N V domain complex with S100A1 versus residue numbers of RAGE V domain (24–128). I_0_ denotes the initial intensity of ^15^N V domain, and I denotes the intensity of ^15^N V domain complex with S100A1. The blue line represents the criterion of selected residues that exhibit significantly decreasing cross-peak signals, and the chosen residues are indicated by red bars. (C) Ribbon representing the structure of V domain and residues that exhibit significantly decreasing cross-peak signals are mapped on the structure in blue.

The experimental method was also used on S100A1 titration with ^15^N-labeled V domain to determine the residues of V domain binding to S100A1. The spectra of HSQC titrations of free and complexed V domain are superimposed in Fig 2A.

A bar graph was constructed by comparing the cross-peak intensities of free V domain to the V domain in complex with S100A1, as shown in Fig 2B.

Residues with significantly lower intensity were chosen for mapping on the 3D structure of the V domain (Fig 2C). The residues K39, R48, W51, K52, L53, D93, F97, K110, and V115 were situated at the B3, B6, and B7 sheets and L3, L4, and L8 loops of the RAGE V domain as binding sites to S100A1. Furthermore, the residues were predicted by NACCESS to have an important role in the S100A1-RAGE V domain complex analysis. Overall, labeled residues on each protein exhibited the binding interface of the protein-protein complex.

### 3.3. Structural of S100A1-V domain complex

The structure of the complex containing S100A1 and V domain was acquired using HADDOCK to calculate the protein-protein interactions. AIRs were acquired from the variation in resonance line broadening in the HSQC spectra of S100A1 and the V domain. The residues with line broadening spectra (HSQC) provided constraints for the calculations in HADDOCK as the input parameters. The HADDOCK results generated structures for the heterotetrameric complex of S100A1 and V domain. The structure for Ca^2+^-bound S100A1 was taken from PDB ID: 2LP3. NMR structural coordinates of the V domain were acquired from PDB ID: 2E5E.

**Fig 3:**
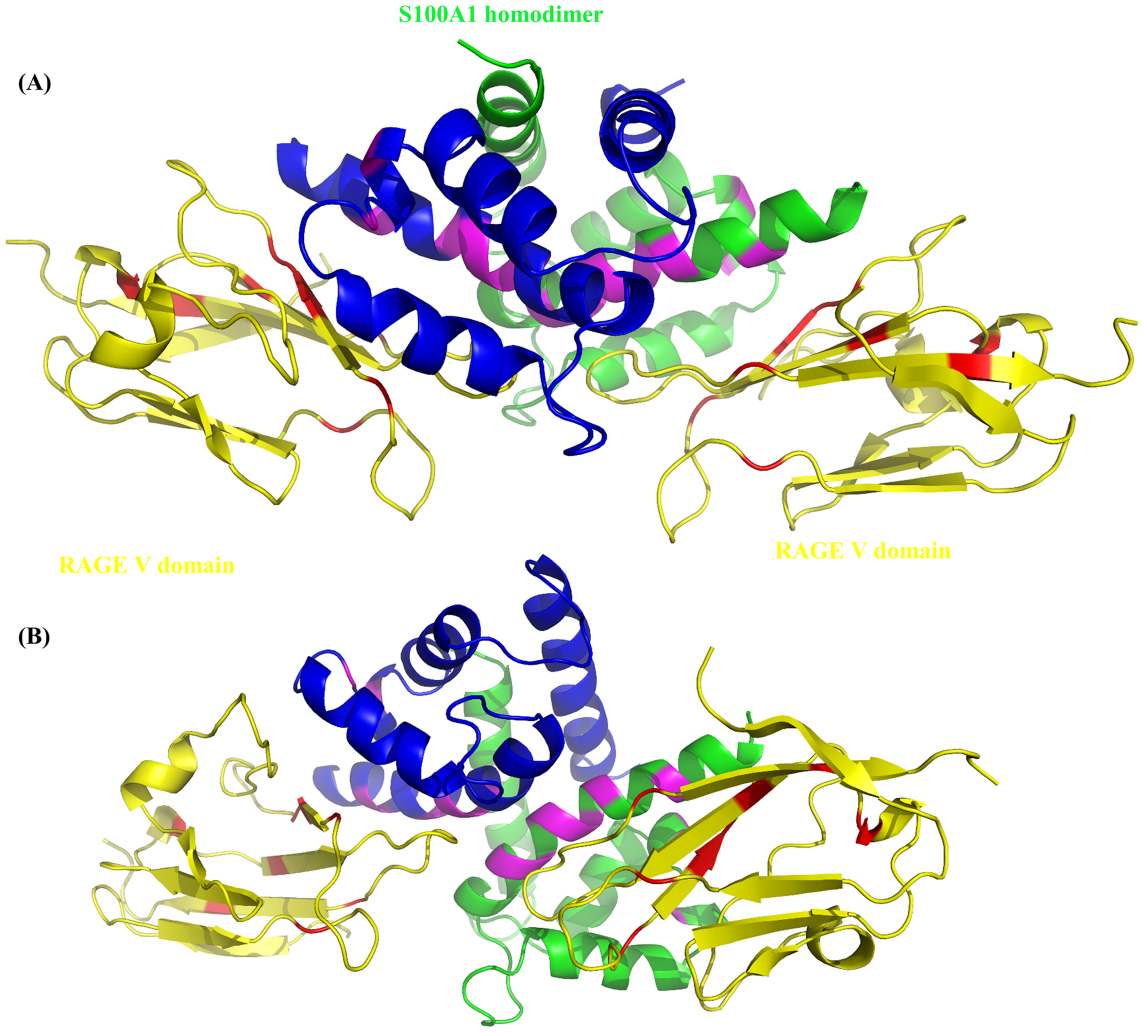
Complex structure of S100A1 and RAGE V domain. (A) Ribbon diagram of the S100A1-V domain complex. Two monomers of S100A1 are shown in green and blue; the two RAGE V domains are shown in yellow. Interacting residues are shown in magenta and red. (B) Ribbon diagram of the S100A1-V domain complex showing with different angle.

Approximately 5000 structural complexes were generated using rigid-body minimization by HADDOCK calculation. The top 200 structures with the lowest energy scores following water refinement were retained for further analysis. The S100A1-RAGE V domain complex is shown in a ribbon diagram (Fig 3).

The S100A1 monomers are shown in green and blue, and the two V domains are shown in yellow. The interacting residues (G43, A47, V76, L77, A79, A80, L81, V83, and N87) are labeled in magenta on S100A1 and in red on the RAGE V domain (K39, R48, W51, K52, L53, D93, F97, K110, and V115). From Ramachandran analysis, the calculated structure of the S100A1-RAGE V domain complex had reasonable bond angles φ and ψ in the stereochemistry of the proteins. The Ramachandran plot (S4 Fig) shows that 91.8% of the complex residues without glycine were in the favored region, and 4 of the complex residues (1.1%) were in the outlier region only.

### 3.4. Binding interface of S100A1 and S100B

**Fig 4:**
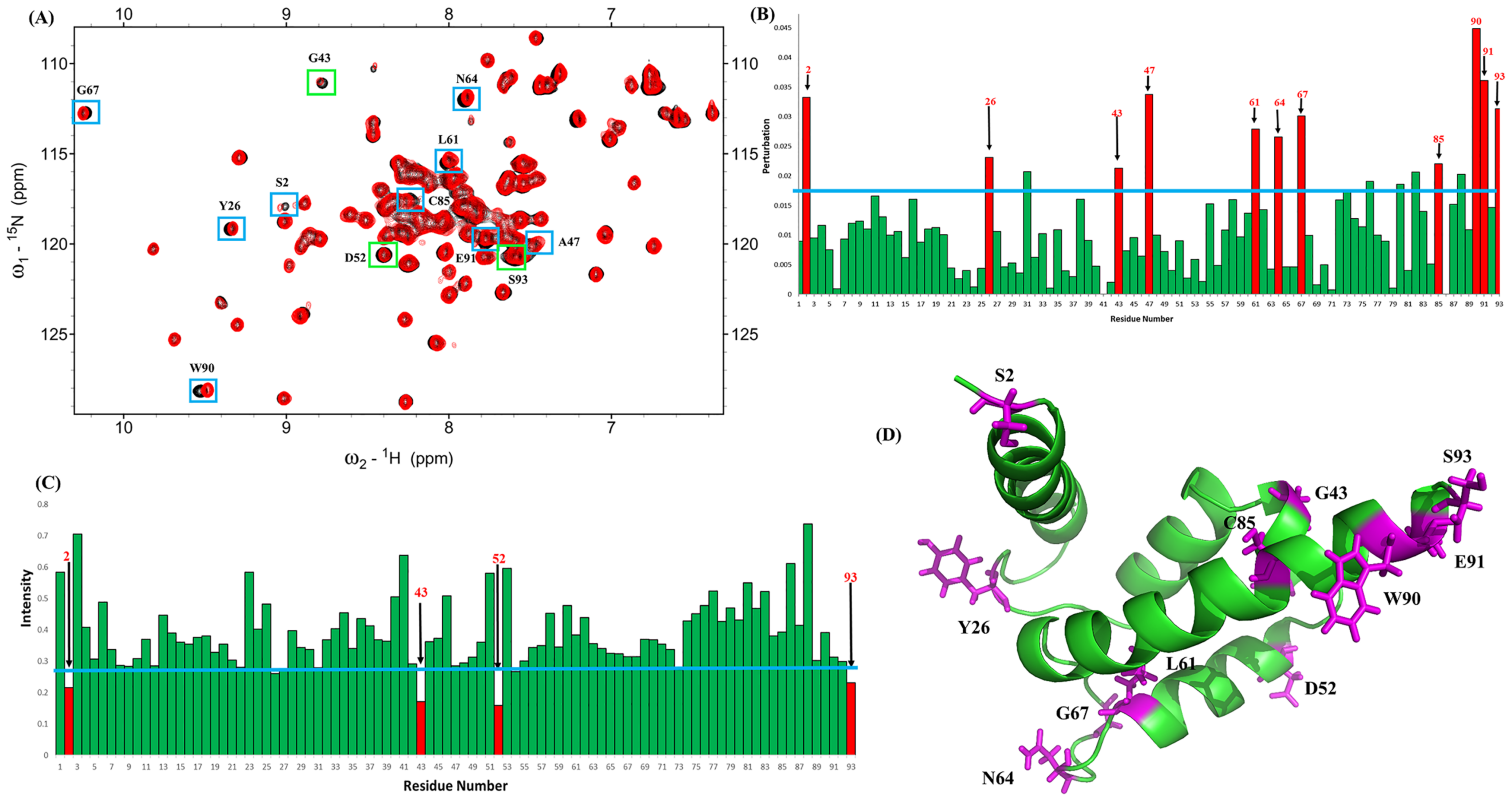
Analysis of the ^1^H–^15^N HSQC spectra of S100A1 in complex with the unlabeled S100B. (A) Overlapped NMR HSQC spectra of ^15^N-S100A1 in black and ^15^N S100A1 complex with unlabeled S100B in a 1:1 ratio (red). Residues displaying significant intensity decreases and perturbed chemical shifts are shown by boxes (green and blue, respectively). (B) Bar plot presenting the changes in cross-peak chemical shift of free-form S100A1 and the S100A1-S100B complex. The blue line shows the threshold of selected residues (red) that display a notable perturbation. (C) Bar plot presenting the decrease in the cross-peak intensity ratio (I/I_0_) of S100A1 and the S100A1-S100B complex. The threshold (blue line) of chosen residues indicates a notably reduced intensity. Selected residues are highlighted in red. (D) Ribbon (stick) diagram of S100A1 in green obtained using the PyMOL program. The residues that exhibit chemical shift perturbations and intensity decreases are shown in magenta.

We used ^1^H-^15^N HSQC spectra to recognize protein-protein binding sites [81,82]. The HSQC of ^15^N S100A1 and ^15^N S100A1 complexed with unlabeled S100B were superimposed (Fig 4A).

The residues between S100A1 and S100B in the interface region were determined by their chemical shift perturbation residues (Fig 4B) and decreased intensities (Fig 4C). At a 1:1 ratio, the signals for the S100A1-S100B complex were observed in intermediate exchange in the NMR time scale (line broadening). These results resulted from the influence of ^15^N-nuclei at the protein interface that formed upon complexation, which led to an obvious decline in the intensities of cross-peaks.

Residues displaying signal reduction and perturbed chemical shifts are shown by yellow and blue boxes, respectively. Cross-peaks with significant perturbation are shown in red (Fig 4B), and the blue line shows the chosen threshold. Residues with perturbed chemical shifts include residues S2, Y26, G43, A47, L61, N64, G67, C85, W90, E91, and S93. Decreasing cross-peak intensities occur for residues S2, G43, D52, and S93 (Fig 4C). The residues were chosen and mapped on the S100A1 3D structure (Fig 4D).

### 3.5. Binding interface of S100B and S100A1

**Fig 5:**
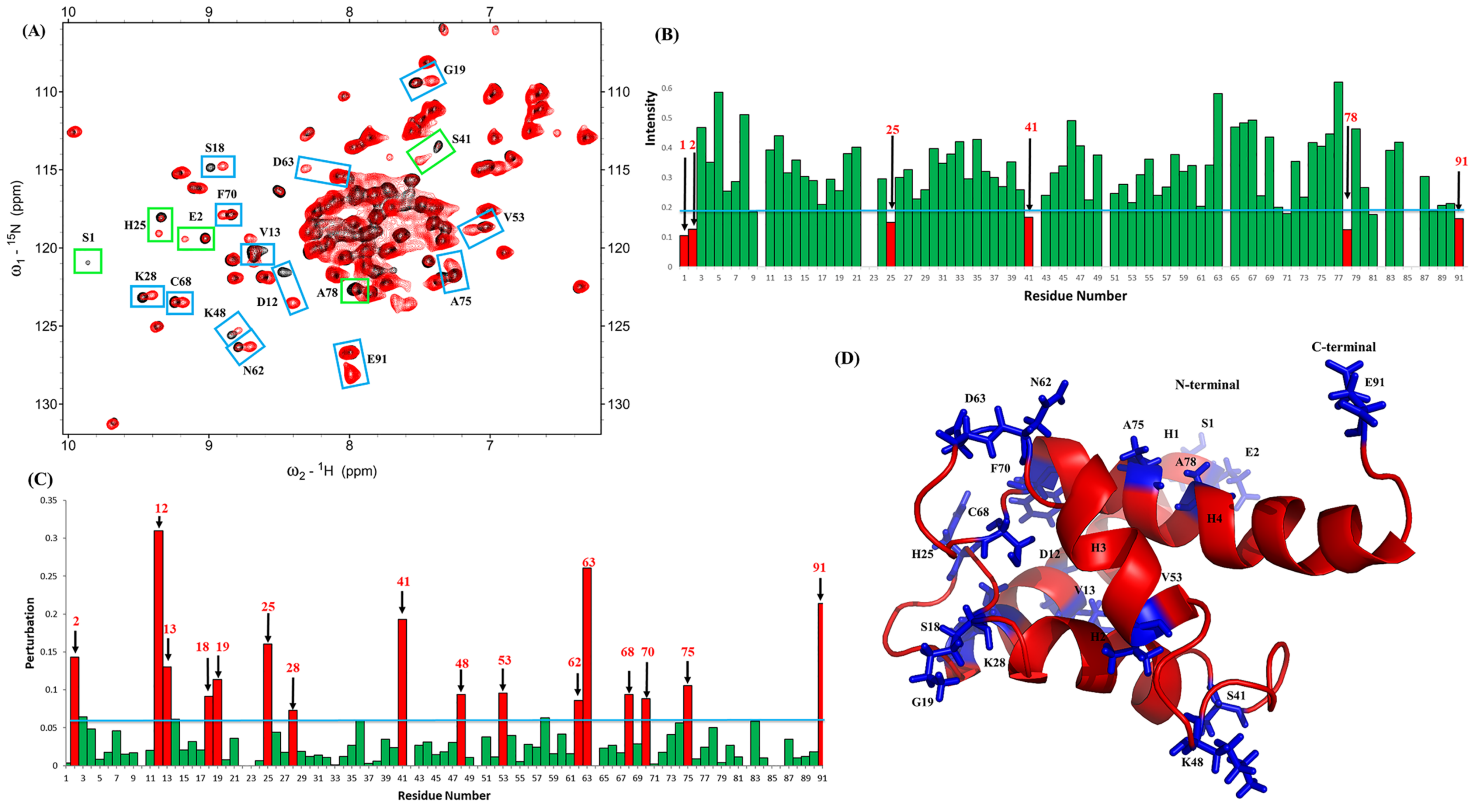
Analysis of the ^1^H–^15^N HSQC spectra of S100B in complex with the unlabeled S100A1. (A) Overlapped NMR HSQC spectra of ^15^N-S100B in black and ^15^N labeled S100B complex with unlabeled S100A1 in a 1:1M ratio (red). Residues displaying significant intensity decreases and those with perturbed chemical shifts are framed by boxes (green and blue, respectively). (B) Bar plot showing the changes in the intensity ratio (I/I0) comparing the ^15^N S100B and the complex of ^15^N S100B with S100A1. The blue line shows the threshold of selected residues that display a notably reduced intensity. (C) Bar plot presenting the changes in chemical shift perturbation comparing free ^15^N S100B and the ^15^N S100B in complex with Ca^2+^-bound S100A1. The blue line shows the threshold of selected residues that display a notable perturbation. (D) Ribbon (stick) diagram of the S100B in red obtained using the PyMOL program. The residues that show chemical shift perturbations and intensity decreases are shown in blue.

For HSQC, the spectrum of the free ^15^N**-**labeled S100B domain was superimposed on the spectrum of the ^15^N-labeled S100B in complex with labeled S100A1 (Fig 5A).

Decreases in the intensities (line broadening) of the cross-peaks were observed, such as those for S1, E2, H25, S41, A78, and E91. Intensity ratios of the free S100B (I_0_) and the complex with unlabeled S100A1 (I) were plotted as a bar graph (Fig 5B). Peaks with significant line broadening are represented in red. The perturbed chemical shifts compared to the free S100B (N_0_, H_0_) and S100A1 (N, H) S100B complex were calculated using the equation. Cross-peaks with significant perturbation are shown in red (Fig 5C). The residues with perturbed chemical shifts included E2, D12, V13, S18, G19, H25, K28, S41, K48, V53, N62, D63, C68, F70, A75, and E91. The residues were chosen and mapped on the 3D structure of the S100A1 domain (Fig 5D).

### 3.6. Structural model of S100A1-S100B complex

The structure of the complex containing S100A1 and S100B was obtained using HADDOCK to calculate the protein-protein interactions. AIRs were acquired from the variation in line broadening and the HSQC spectra of S100A1 and S100B. The residues with line broadening spectra (HSQC) provided constraints for the calculations in HADDOCK as input parameters. The HADDOCK results generated structures for the heterodimeric complex of S100A1 and S100B. The structure for Ca^2+^-bound S100A1 was taken from PDB ID: 2LP3. The NMR structure of S100B was acquired from PDB ID: 1UWO.

**Fig 6:**
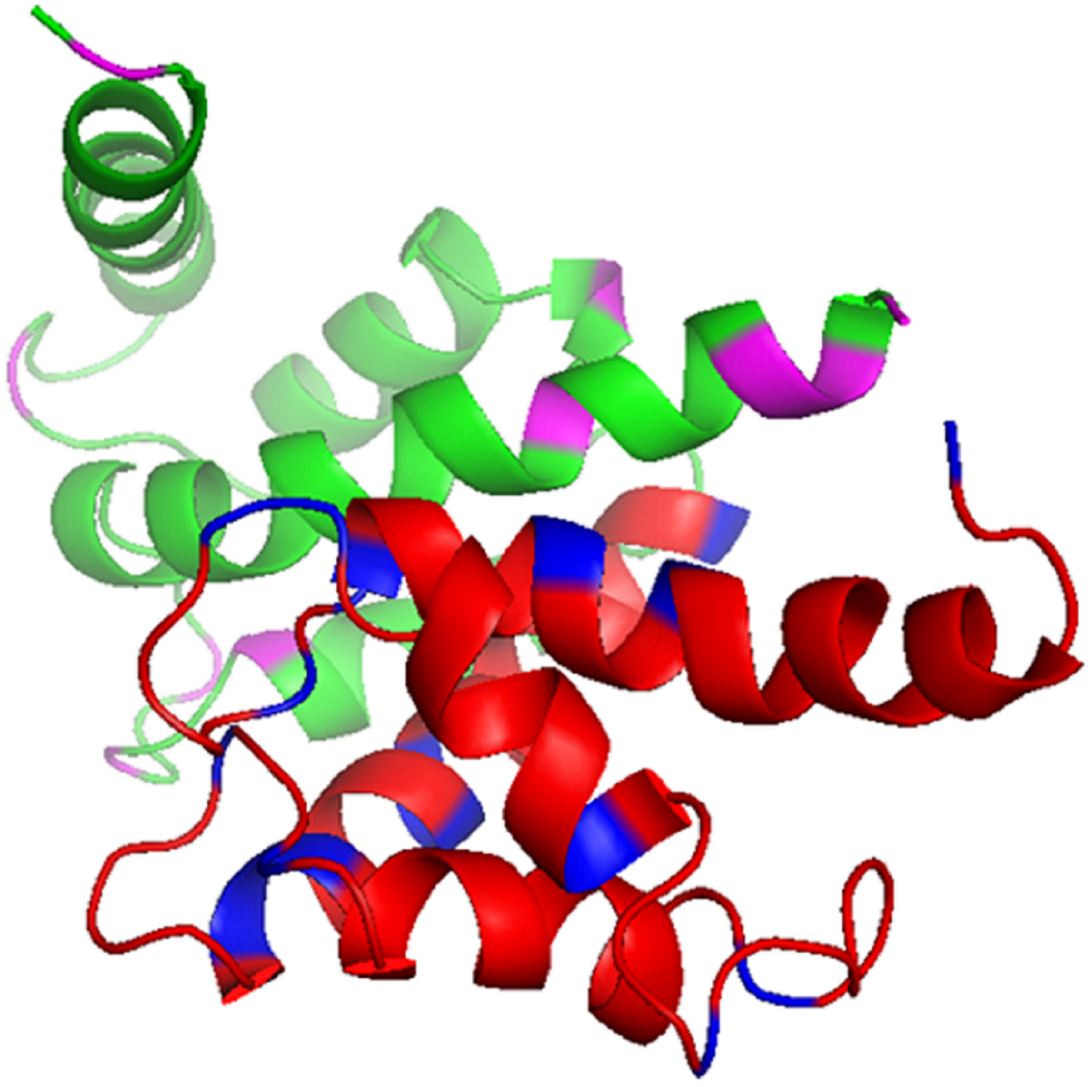
Ribbon diagram of the S100A1-S100B complex. The S100A1 is shown in green, the S100B is shown in red. The interacting residues of S100A1 (magenta and the S100B (blue) are shown as sticks.

Approximately 5000 structural complexes were generated using rigid-body minimization by HADDOCK calculation. The best 200 structures with the lowest energy scores following water refinement were retained for further analysis. The S100A1-S100B complex is shown in a ribbon diagram in Fig 6. The S100A1 is shown in green and the S100B is shown in red. The interacting residues (S2, Y26, G43, A47, D52, L61, N64, G67, C85, W90, E91, and S93) are labeled in magenta on S100A1 and in blue on the S100B (S1, E2, D12, V13, S18, G19, H25, K28, S41, K48, V53, N62, D63, C68, F70, A75, A78, and E91). Ramachandran analysis demonstrated that 85% of the residues are located at the most favored region of the Ramachandran plot (S5 Fig).

### 3.7. Determination of the dissociation constant of the S100A1-V domain complex

According to the changes in the chemical shift perturbation, the dissociation constant (K_d_) can be determined by analysis of the NMR HSQC titration spectrum [79]. We selected the cross-peaks that changed dramatically in the HSQC spectra of ^15^N S100A1 titrated with unlabeled RGAE V domain. Residues V76, L77, A79, A80, L81, V83, and N87 were picked for our ideal model using the following equation:

$$\Delta \delta_{obs}=\Delta \delta_{\text{max}}\times \left\{\left[\left({\left[\text{P}\right]}_{\text{t}}+{\left[\text{L}\right]}_{\text{t}}+{\text{K}}_{\text{d}}\right)-\sqrt{{\left({\left[\text{P}\right]}_{\text{t}}+{\left[\text{L}\right]}_{\text{t}}+{\text{K}}_{\text{d}}\right)}^{2}+4{\left[\text{P}\right]}_{\text{t}}{\left[\text{L}\right]}_{\text{t}}}\right]÷2{\left[\text{P}\right]}_{\text{t}}\right\}$$ (2)

In this equation, Δδ_obs_ is the change observed in the shift between the free form and bound form at a molar ratio 1:1. Δδ_max_ is the maximum shift change at a molar ratio of 1:2, and [P]t and [L]t are the total concentrations of protein and ligand (RAGE V domain) in solution. These equations allow us to calculate K_d_ from the calculated shift at different concentrations of protein.

**Fig 7:**
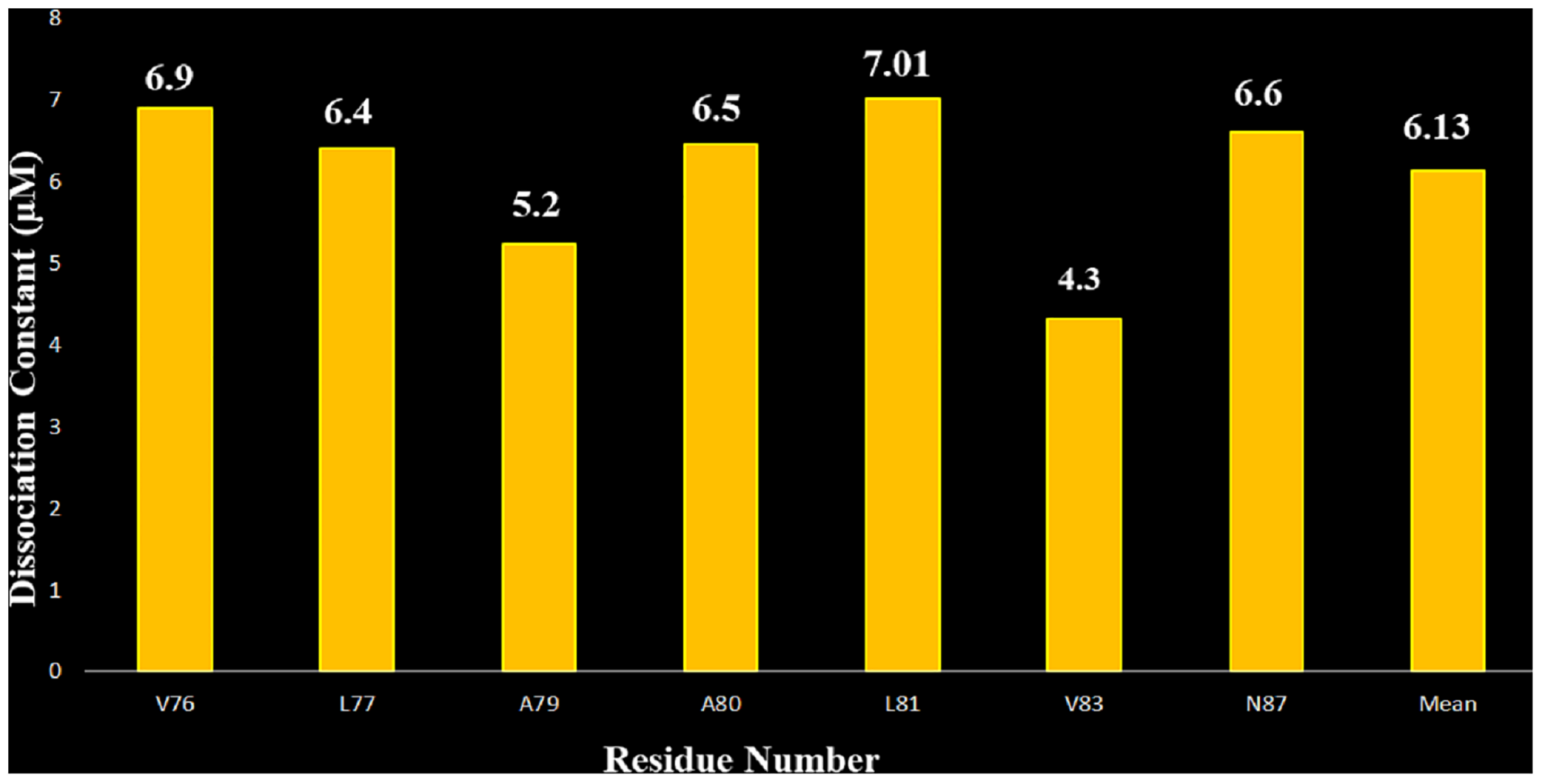
Bar graph of the selected residues observed in ^15^N S100A1 HSQC titrations with RAGE V domain spectrum versus the corresponding dissociation constant K_d_ with a 1:1 molar ratio. The mean value of K_d_ is about 6.13 ± 1.29 μM.

**Fig 8:**
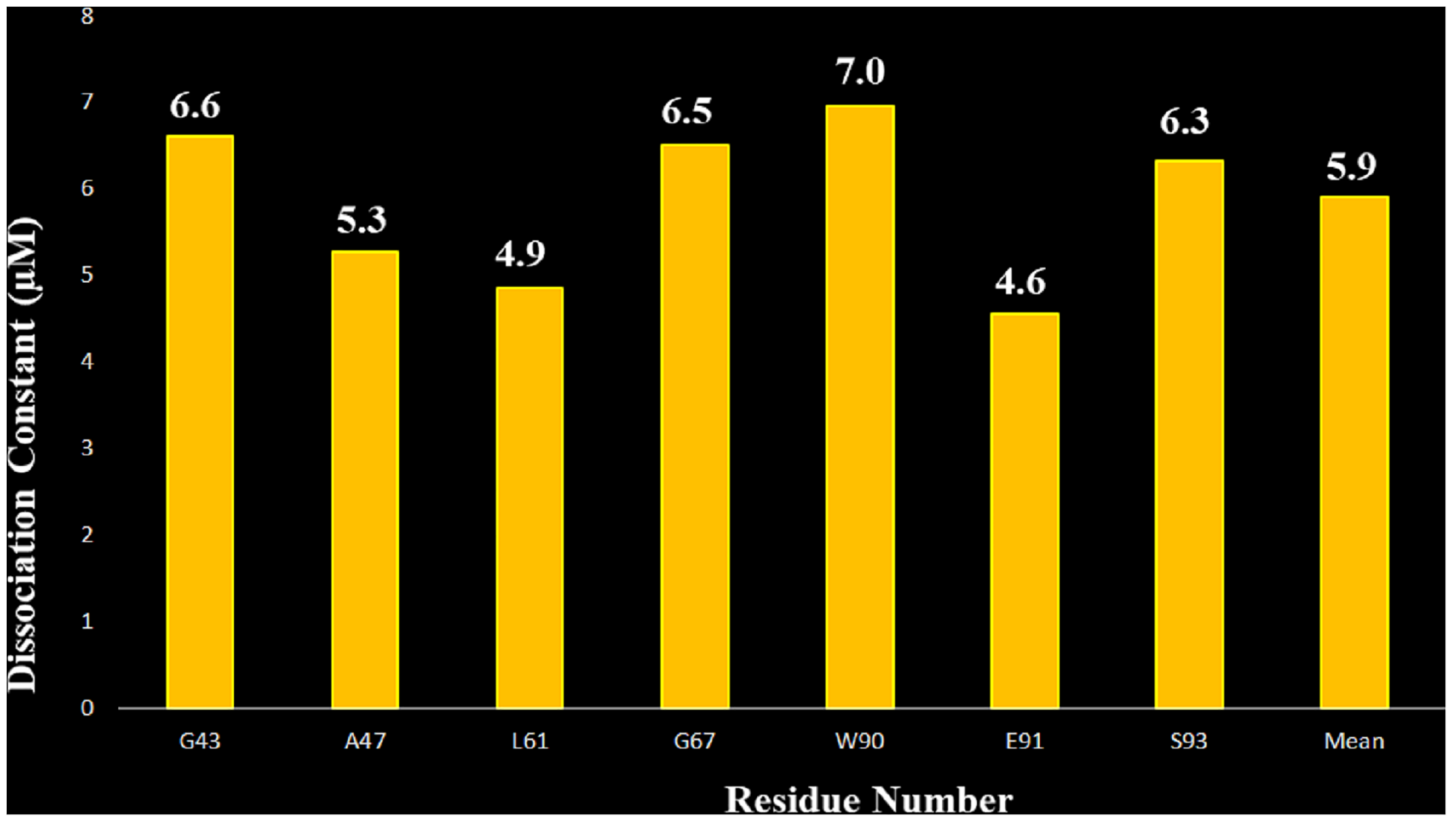
Bar graph of the selected residues observed in ^15^N S100A1 HSQC titrations with S100B spectrum versus the corresponding dissociation constant K_d_ with a 1:1 molar ratio. The mean value of K_d_ is about 5.9 ± 1.20 μM.

From the HSQC titration we have compared the chemical shifts of the free (S100A1, S100B) and the complex (S100A1-V domain, S100A1-S100B in molar ratio 1:0.66 and 1:1), shift of 1:0.66 is taken as observed and 1:1 as maximal for only those residues that shows chemical shift perturbation. Then, we calculated the K_d_ with assessing the uncertainty for each protein complex such that the K_d_ for S100A1 and V domain is 6.13±1.29 μM (Fig 7) and K_d_ 5.9±1.20 μM (Fig 8) for S100B.

### 3.8. Fluorescence method to determine the dissociation constant of the S100A1-S100B complex

**Fig 9:**
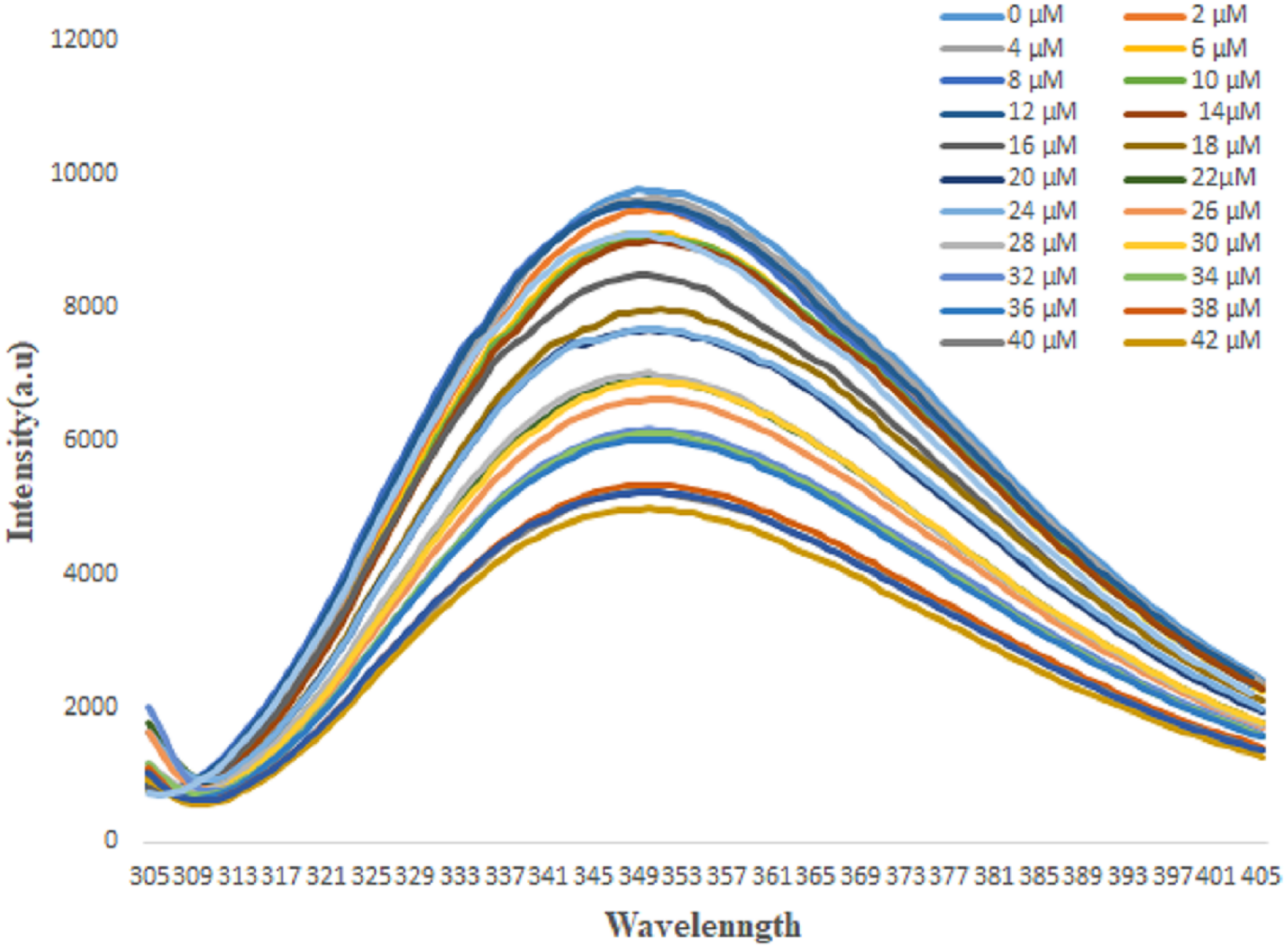
Emission spectra of S100A1 fluorescence titrations exhibiting decreasing fluorescence intensities with increasing concentration of S100B at the μM level.

**Fig 10:**
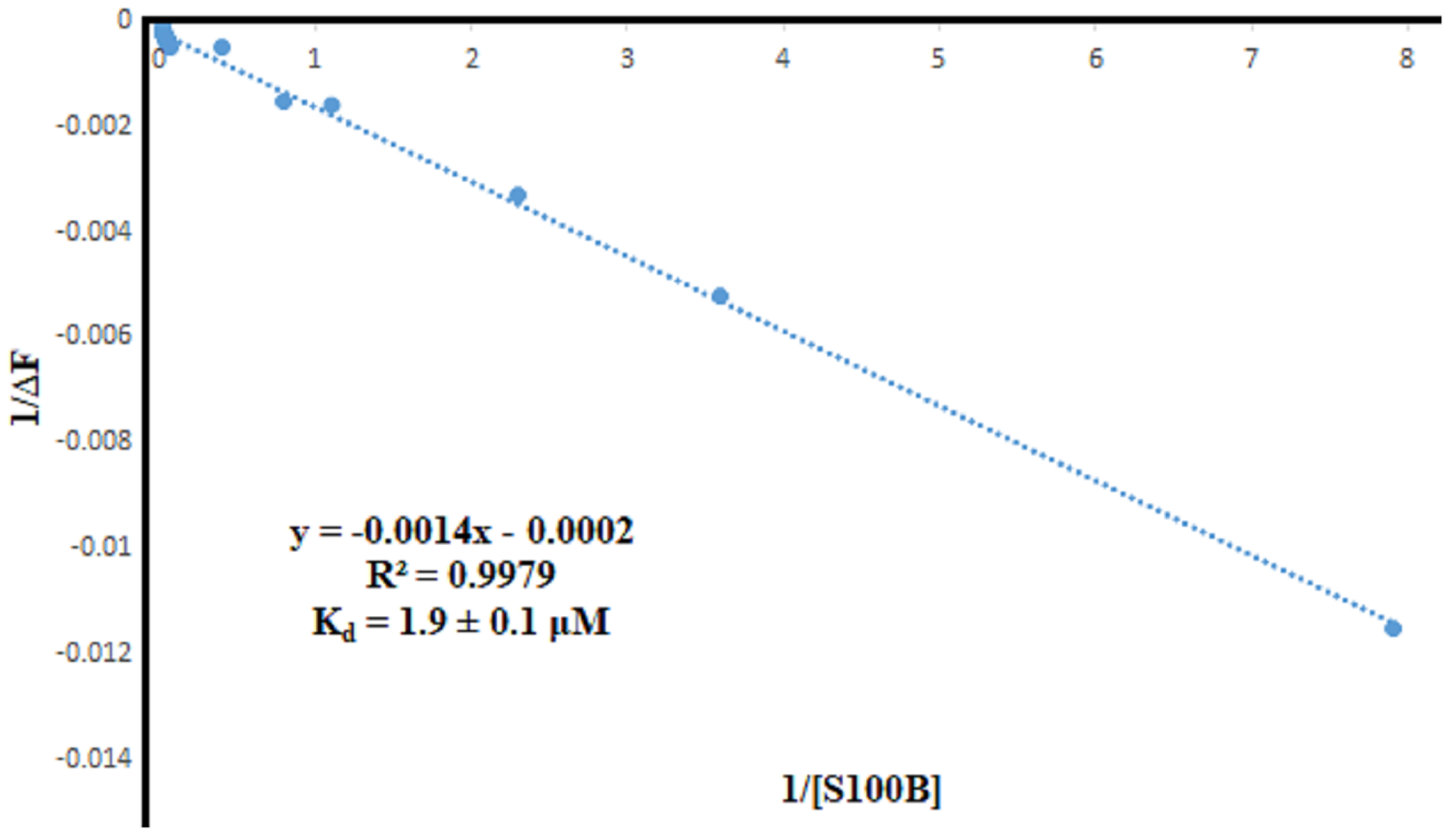
Curve with fluorescence intensity changes versus S100B concentrations obtained at a wavelength of 351 nm. K_d_ is calculated as 1.9 ±0.1 μM from Eq 4 [83].

All of the fluorescence experiments were performed under the same buffer conditions. The S100A1 has one tryptophan residue that could be excited and emit fluorescence. Fluorescence experiments were performed using an F-2500 fluorescence spectrophotometer (Hitachi). We used an excitation wavelength of 295 nm. S100A1 has a maximum emission band at a wavelength of 351 nm. We detected the emission wavelengths in the range of 305–404 nm. Increasing concentrations of S100B of 0 to 42 μM were added at increments of 2 μM to the S100A1 (Fig 9), which had a concentration of 3.50 μM. The data acquired from both fluorescence experiments (Fig 10) were plotted as 1/[S100B] versus 1/ (I _ I_0_).

We used the following equations to a fit linear curve in the Origin program, which was used to calculate the dissociation constant of the two samples [84]:

$$\frac{1}{\left(\text{I}-{\text{I}}_{0}\right)}=\frac{1}{\left({\text{I}}_{1}-{\text{I}}_{0}\right)}+\frac{{\text{K}}_{\text{d}}}{\left({\text{I}}_{1}-{\text{I}}_{0}\right)}\times \frac{1}{\left[\text{S100B}\right]}$$ (3)

$$\Delta \text{F}=\frac{\Delta {\text{F}}_{\text{max}}\times \left[\text{S100B}\right]}{{\text{K}}_{\text{d}}+\left[\text{S100B}\right]}$$ (4)

where I_0_, I, and I_1_ are the emission intensities in the absence of S100B, in the middle concentration of S100B, and in the maximum concentration of S100B, respectively. F is the difference in fluorescence intensity between I and I_0_. *F*max is the maximal fluorescence change, and K_d_ is the dissociation constant.

### 3.9. Functional assay

A cell proliferation assay can be used to characterize the downstream stimulation of functions mediated by human S100A1. However, the assay is critically affected by disruptions to the protein interaction. It has been reported that RAGE was highly expressed in colorectal cancer tissues, and was associated with increased microvessel density. SW480 cell line is a RAGE highly expressed colorectal cancer cell line. Knockdown of RAGE expression by the shRNAs specifically against RAGE gene inhibits colorectal cancer cell invasion and suppresses angiogenesis, indicating that RAGE pathway is critical in the malignancy of SW480 cells [85]. Thus, we chose SW480 cells for our functional assay and treated them for 48 h with S100A1. Viable cells were analyzed using a WST-1 assay.

**Fig 11:**
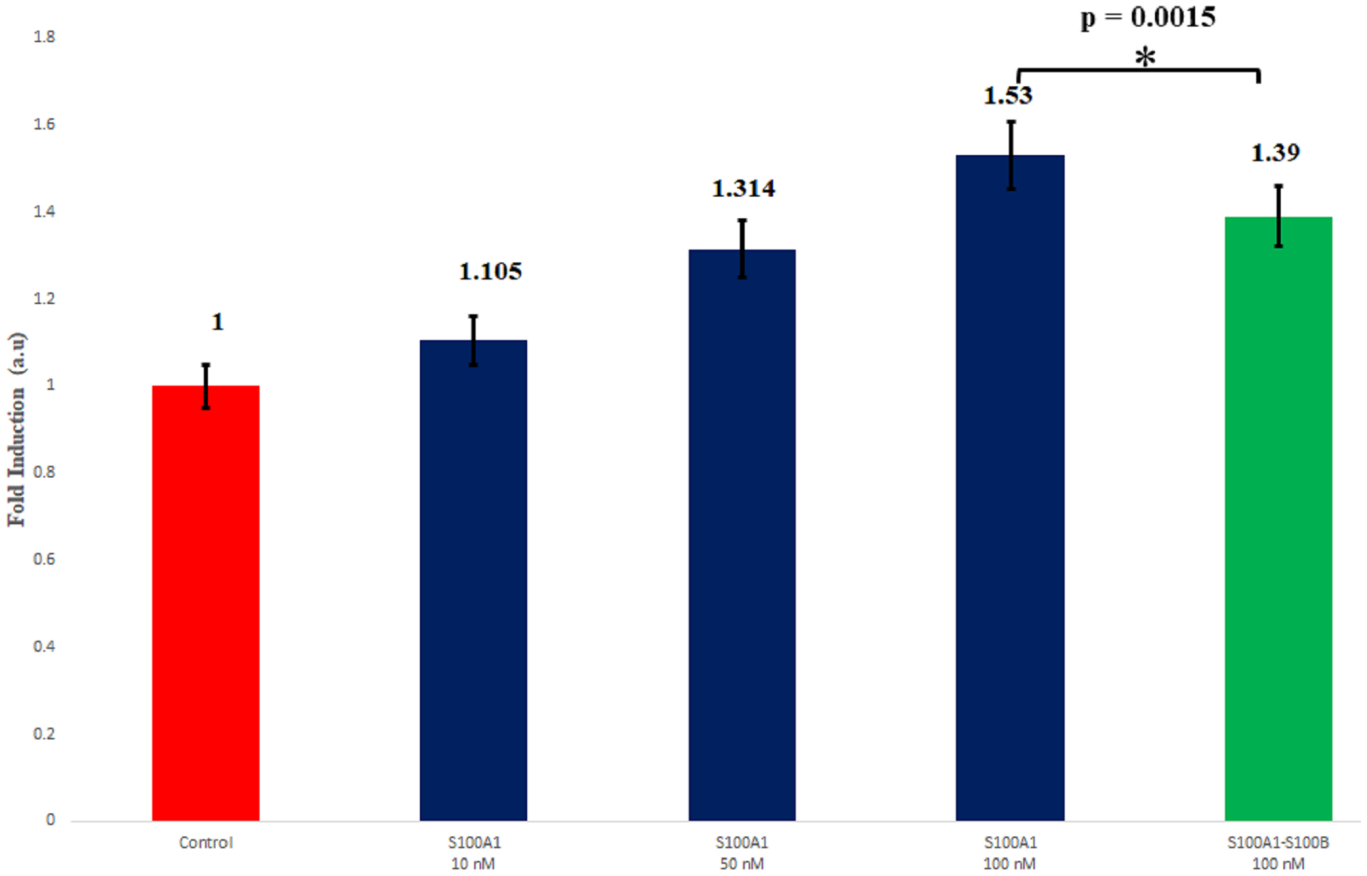
Analysis of WST-1 assays. A. SW-480 cells were treated with 10 nM, 50 nM, or 100 nM S100 A1 (blue); 100 nM S100 A1 + 100 nM S100B (green). Cell proliferation was analyzed using a WST-1 assay. The relative cell counts following treatment with S100A1 are plotted as the fold induction with serum-free media as the corresponding controls (red). The data are expressed as the mean ± SD from 4 independent experiments.

Serum-starved SW-480 cells were grown under treatment with increasing concentrations of S100A1 (10, 50, and 100 nM). As the concentration of S100A1 increased to 100 nM, a 1.53-fold increase was observed in the viable cell count compared to the serum-free control (Fig 11 Lane 4). The proliferative activity of the SW480 cells decreased cell numbers to 1.39-fold when 100 nM S100B was added (Fig 11, Lane 5). Comparing these two groups, the p-value was 0.0015 by t-test, indicating that addition of S100B significantly decreased S100A1-induced cell proliferation. In the study, we found that S100A1 protein directly interacted with RAGE V domain *in vitro* and extracellular treatment of S100A1 protein significantly increased cell proliferation in a dose-dependent manner in SW480 cells. These results suggested that S100A1-induced cell proliferation, at least in part, might be via RAGE pathway, due to the highly expressed RAGE in SW480 cells. However, other signaling pathways might be also involved in S100A1-induced cell proliferation in SW480 cells. A previous study showed that S100B specifically interacted with the RAGE V and C1 domains, and activated PI3K/AKT and and NF-kappaB pathways, leading to increase of cell numbers/viability in human SH-SY5Y neuroblastoma cells [60] Our results demonstrated that S100A1 protein directly interact S100B protein *in vitro*, and extracellular addition of S100B protein significantly decreased S100A1-induced cell proliferation in SW480 cells, suggesting that the S100A1-S100B hetero dimer might has lower activity towards RAGE rather than S100A1 homo dimer. These results suggest that S100B can indeed influence the binding between S100A1 and the V domains, leading to inhibition of S100A1-induced cell proliferative activity.

## 4. Conclusion

S100 proteins are secreted in an autocrine, endocrine, and paracrine manner, which can produce a sufficient concentration to influence cellular signaling in different cell types. We found the binding site of S100A1 and the V domain in a tetrameric complex and demonstrated their key role in the induction of cell proliferation using ^1^H-^15^N HSQC experiments. The characterization studies on S100A1 and the V domain provided the dissociation constant (K_d_) via NMR spectroscopy. The constant was in the range of 6–7 μM, indicating a reasonably strong interaction. Moreover, NMR titration experiments also suggested that binding occurred on the surface of the S100A1-RAGE V domain complex. We conclude that S100A1 binds to the V domain through hydrophobic residues, providing specific recognition for the S100A1–RAGE V domain interaction.

**Fig 12:**
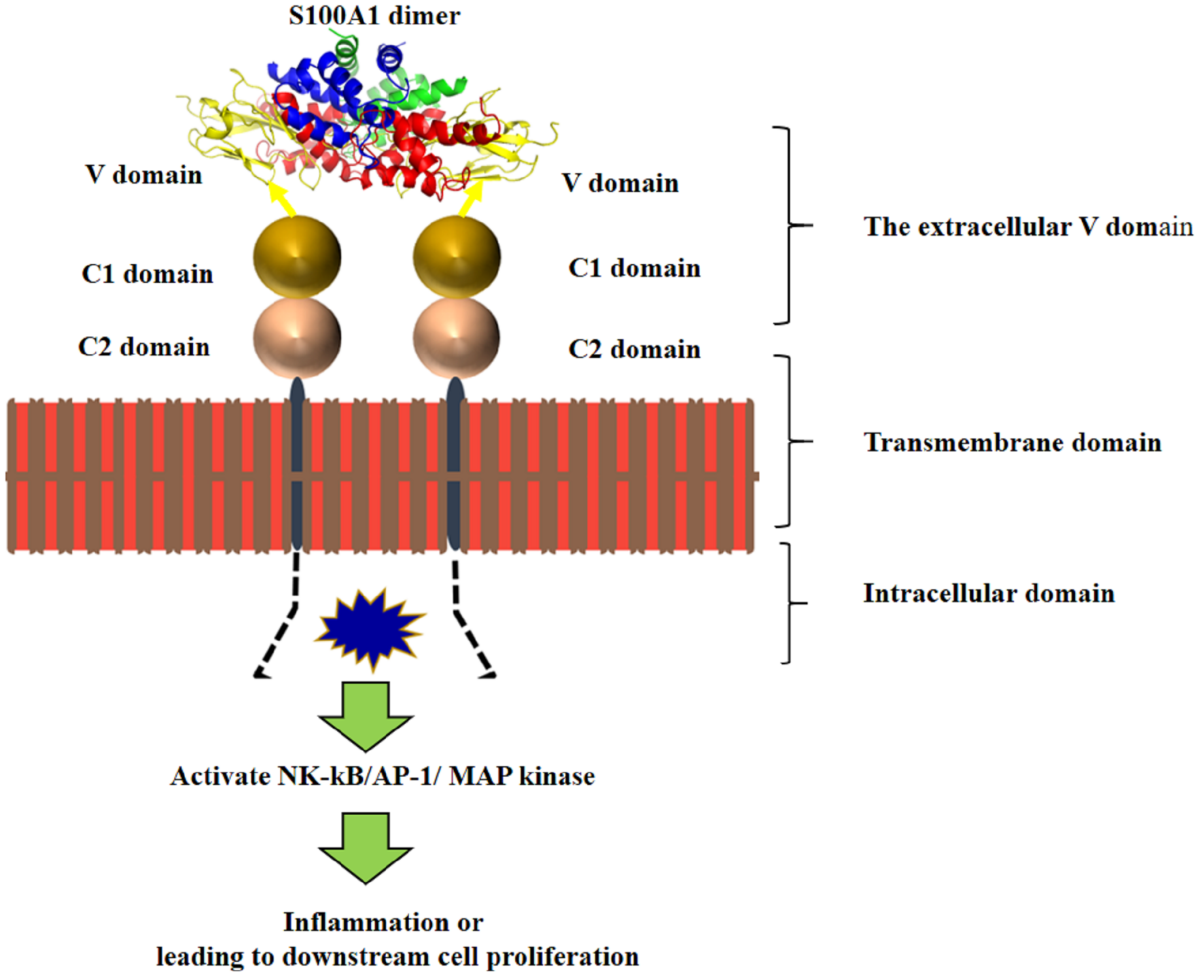
Hypothetical mechanism of the S100A1-RAGE signaling pathway. The extracellular V domain of the RAGE protein binds to the S100A1 dimer, facilitating intracellular dimerization of the cytoplasmic domain. This activates a signaling cascade (MAP kinase, NF-κB, and AP-1), leading to downstream cell proliferation or inflammation.

We also calculated a model of the S100A1-V domain heterotetramer complex using the HADDOCK program. A pathway for S100A1-RAGE signaling was proposed in this study (Fig 12). The homodimer of S100A1 first binds to the V domain, which results in RAGE dimerization. The cytoplasmic domain becomes activated for autophosphorylation, leading to a signaling cascade that subsequently regulates cell proliferation. We also demonstrated that S100B binds to S100A1.

To characterize the binding between S100A1 and S100B, the interacting residues were determined by NMR titration experiments. Coincidentally, both the RAGE V domain and S100B bind to S100A1 in the same region. These results indicate that S100B could play a crucial role as an antagonist in blocking the interaction between S100A1 and RAGE V domains. Further studies are required to design S100B analogs that could potentially act as efficient blockers that disrupt the S100A1-RAGE V domain pathway for the treatment of various human cancers or inflammation diseases.

**Fig 13:**
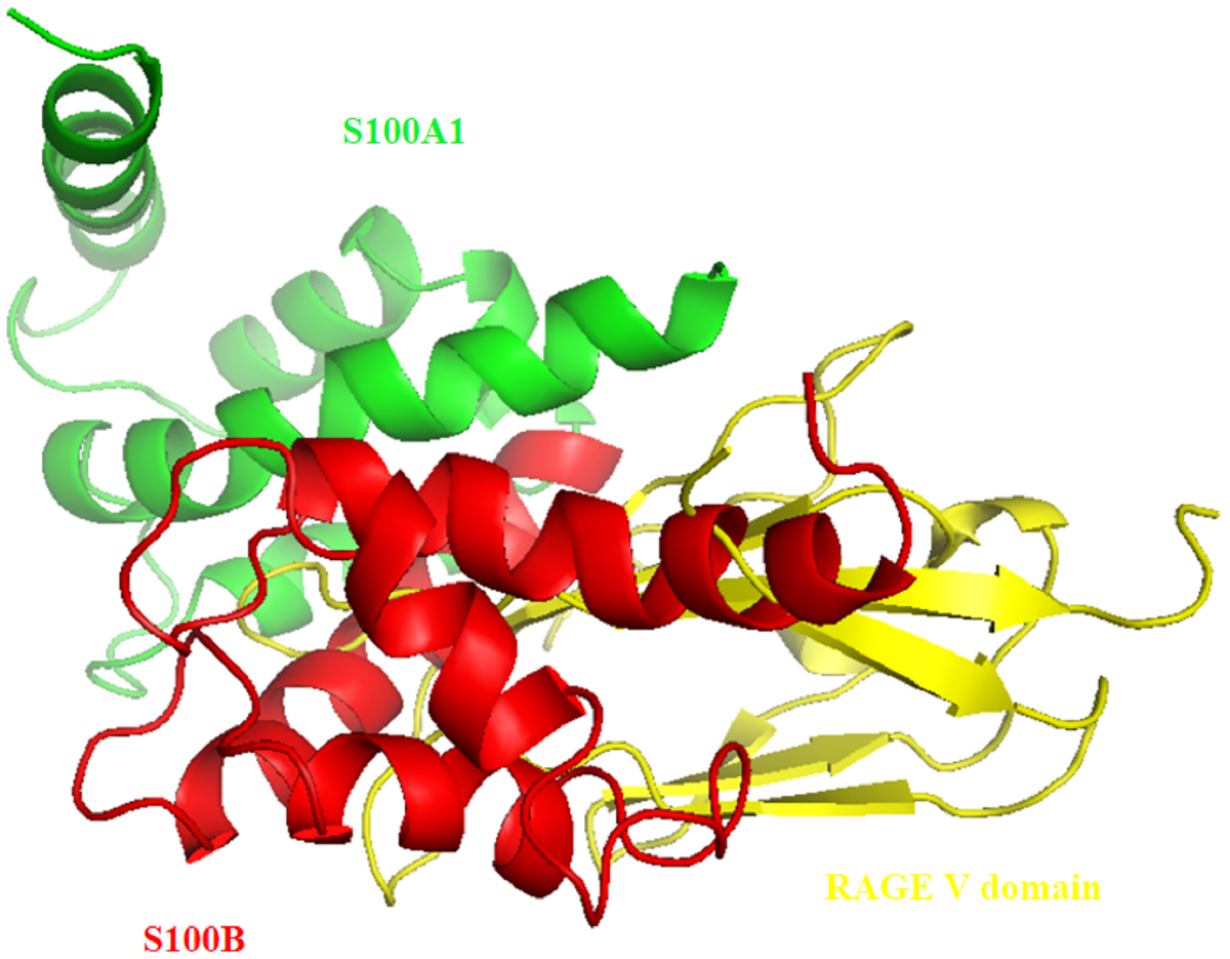
Superimposition of the complex between S100A1 (monomer in green) and the V domain (yellow), with the complex between S100A1 and S100B (red). It is demonstrated that S100B blocks the interaction between S100A1 and the V domain.

The following two structural complexes were superimposed: (1) S100A1 complexed with a RAGE V domain, and (2) S100A1 complexed with S100b molecules (Fig 13). The S100A1 subunits are shown in blue and green, the two RAGE V domains are shown in yellow, and S100B molecules are shown in red.

It is clear from the figures that the S100B molecule block the interaction between S100A1 and the V domain. The structural model of this complex could be beneficial for improving antagonists and could help in protein design efforts targeting S100A1 and the V domain.

## Supporting information

S1 Fig SDS-PAGE band for purified S100A1 protein showing molecular weight of 10.5 kDa. (TIF)

S2 Fig SDS-PAGE band for purified RAGE V domain protein showing molecular weight of 12.7 kDa. (TIF)

S3 Fig SDS-PAGE band for purified S100B protein showing molecular weight of 10.7 kDa. (TIF)

S4 Fig Ramachandran plot statistics of the complex of S100A1 with RAGE V domain by PROCHECK analysis. 91.8% of residues are in the favored area, 7.1% are in the allowed area, and 1.1% are in the disallowed region.
(TIF)

S5 Fig Ramachandran plot statistics of the complex of S100A1 with S100B by PROCHECK analysis. 85% of residues are in the favored area, 12.20% are in the allowed area, and 2.8% are in the disallowed region.
(TIF)
