# Supplementary figures and images for: S100B as an antagonist to block the interaction between S100A1 and the RAGE V domain
Source: PLoS One. 2018 Feb 14;13(2):e0190545. doi: 10.1371/journal.pone.0190545 (PMC5812564; doi:10.1371/journal.pone.0190545)

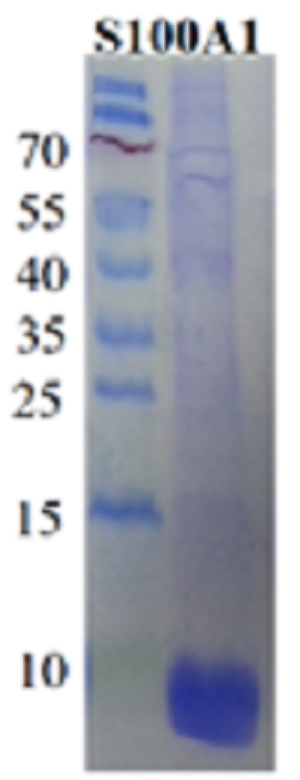

Supplement: S1 Fig — (TIF) [file pone.0190545.s001.tif]

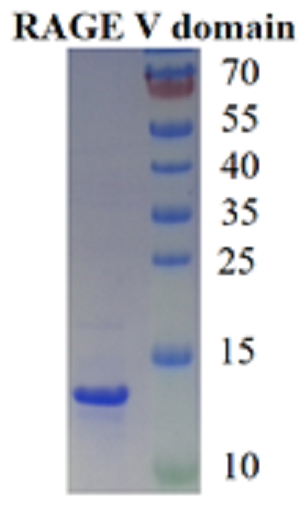

Supplement: S2 Fig — (TIF) [file pone.0190545.s002.tif]

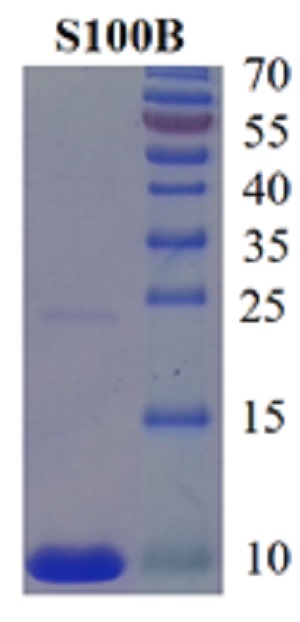

Supplement: S3 Fig — (TIF) [file pone.0190545.s003.tif]

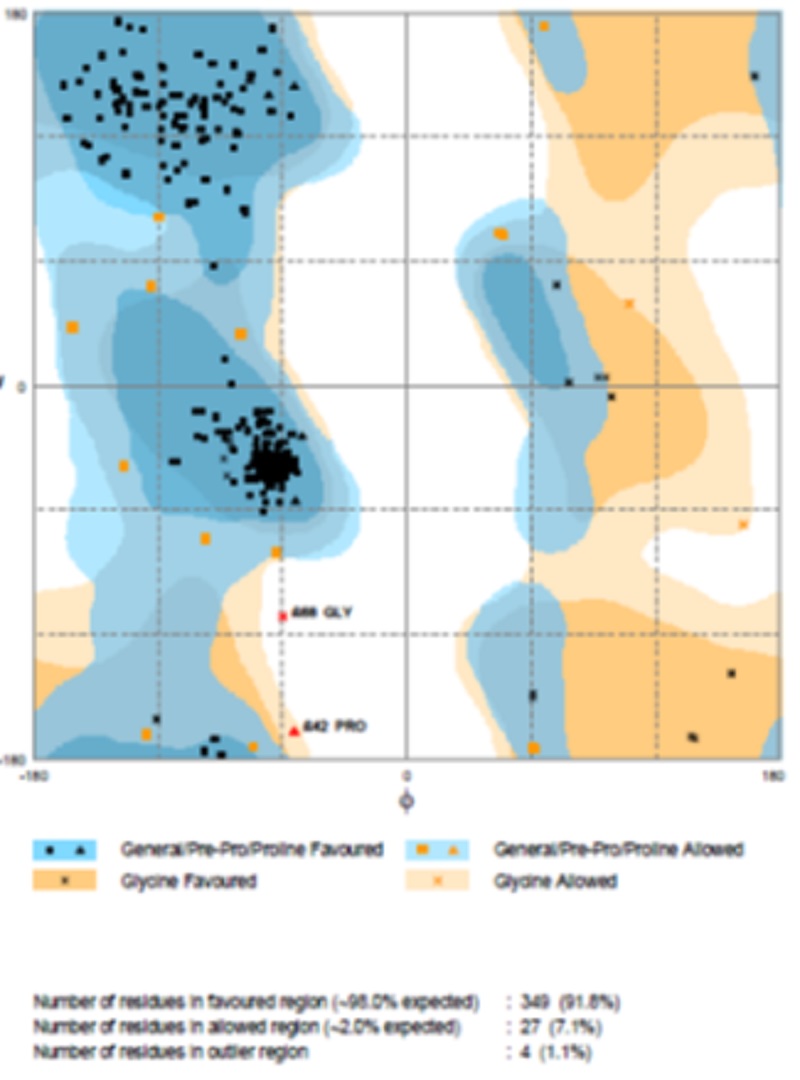

Supplement: S4 Fig — 91.8% of residues are in the favored area, 7.1% are in the allowed area, and 1.1% are in the disallowed region. (TIF) [file pone.0190545.s004.tif]

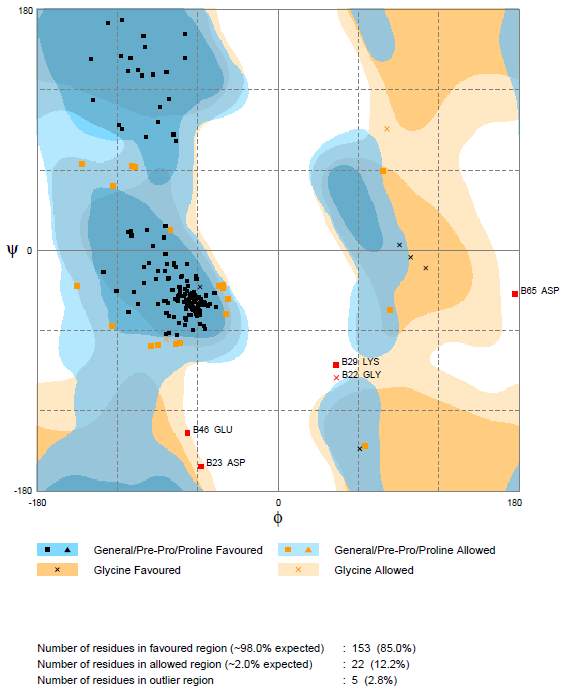

Supplement: S5 Fig — 85% of residues are in the favored area, 12.20% are in the allowed area, and 2.8% are in the disallowed region. (TIF) [file pone.0190545.s005.tif]
